# Psychological Distress, Anxiety, and Academic Self-Efficacy as Predictors of Study Satisfaction Among Peruvian University Students During the COVID-19 Pandemic

**DOI:** 10.3389/fpsyg.2022.809230

**Published:** 2022-04-25

**Authors:** Renzo Felipe Carranza Esteban, Oscar Mamani-Benito, Tomás Caycho-Rodriguez, Susana K. Lingán-Huamán, Percy G. Ruiz Mamani

**Affiliations:** ^1^Facultad de Humanidades, Grupo de Investigación Avances en Investigación Psicológica, Universidad San Ignacio de Loyola, Lima, Peru; ^2^Escuela de Psicología, Facultad de Derecho y Humanidades, Universidad Señor de Sipán, Chiclayo, Peru; ^3^Facultad de Ciencias de la Salud, Universidad Privada del Norte, Lima, Peru; ^4^Facultad de Ciencias de la Salud, Universidad Privada San Juan Bautista, Lima, Peru

**Keywords:** psychological distress, anxiety, academic self-efficacy and study satisfaction, predictive analysis, university students, Peru

## Abstract

The objective of this research study was to determine if psychological distress, anxiety, and academic self-efficacy predict satisfaction with studies in Peruvian university students during the COVID-19 pandemic. A cross-sectional and predictive design was used, in which 582 Peruvian university students participated, 243 men and 339 women, between the ages of 16 and 41. Student’s *t*-statistics were used to analyze the differences in scores of psychological distress, anxiety, academic self-efficacy, and satisfaction with studies based on the sex of the participants, Pearson’s *R* was used for the analysis of correlations between variables, and multiple linear regressions were used to evaluate the predictive model. In the analyses, the significance level was set at 0.05. The results show that men have higher levels of psychological distress, anxiety, and academic self-efficacy than women do (*p* < 0.01); high levels of psychological distress correlate with high levels of anxiety (*r* = 0.580, *p* < 0.01) and low levels of satisfaction with studies (*r* = –0.178, *p* < 0.01) and academic self-efficacy (*r* = −0.348, *p* < 0.01); high levels of anxiety correlate with low levels of satisfaction with studies (*r* = −0.122, *p* < 0.01) and academic self-efficacy (*r* = –0.192, *p* < 0.01); and high levels of academic self-efficacy correlate with high levels of satisfaction with studies (*r* = 0.429, *p* < 0.01). Academic self-efficacy was also found to predict satisfaction with studies (β = 0.429, *p* < 0.01). This concludes that, although there are significant correlations between psychological distress, anxiety, academic self-efficacy, and satisfaction with studies, academic self-efficacy is the variable that most predicts satisfaction with studies in Peruvian university students.

## Introduction

Today’s society demands that students graduate from universities with high professional skills (Rodrigo, 2016). This poses certain challenges in scenarios where virtual learning is applied (Durán et al., 2015), as was the case during the COVID-19 pandemic (Britez, 2020).

Because of the imposition of social restriction measures and lockdown to reduce the rate of infections (Peña-Otero et al., 2020) and to prevent overloading health systems (Rodríguez et al., 2020), sectors such as education have had to adapt and propose new strategies to continue the teaching–learning process without lowering educational standards (Bayham and Fenichel, 2020; Van and Parolin, 2020). In the case of Latin America, given the rapid growth in the infection rate (Barboza-Palomino et al., 2020; Gallegos et al., 2020), universities had had to establish virtual platforms based on the use of educational technologies to continue with the academic school year (Hernandez et al., 2020). These changes forced university students to face challenges, such as having to adapt to online education (Chau and Saravia, 2014) and self-regulate their learning (Alegre, 2015).

Even before the pandemic, several studies had reported that university populations are one of the development groups most prone to conflict, which is common during middle and late stage adolescence (Balanza et al., 2009). Primarily, manifestations of stress (Phinder-Puente et al., 2014), anxiety (Reyes et al., 2017), depression, suicidal behavior (Micin and Bagladi, 2011), and other psychological disorders (De Jesus et al., 2019) have been recurrently reported in this population. The impact of the pandemic on higher education study experience has been serious (Costa and Carvalho-Filho, 2020), since traditional education is not the same as learning via conference calls and online exercises (Connor et al., 2020), especially for those who need training in specialized laboratories (Warhadpande et al., 2020), where case virtual classes can hardly replace hands-on learning (Aquino-Canchari and Medina-Quispe, 2020).

Based on what has been described, it is urgent to reflect on satisfaction with studies in university students during the health emergency. Study satisfaction is defined as a positive assessment that an individual makes when comparing their ambitions with what they had actually achieved, a fact that in the academic field is understood as the enjoyment and sense of well-being in the experiences lived (Dominguez-Lara and Campos-Uscanga, 2017), which are precisely the driving force of the learning experience at higher educational levels.

A theoretical model that has proven useful when studying the processes that lead students to feel satisfied with their learning experience is self-determination theory (SDT, Tomás and Gutiérrez, 2019). This is interpreted as a motivational approach that describes the educational circumstances in which students experience enjoyment and well-being (Tarek and Hubbard, 2015). Studies carried out in this field show that there are three psychological conditions necessary toward feeling satisfied with the educational environment (Wang et al., 2019): autonomy (the experience of freedom of choice in learning), competence (perception of self-efficacy and the ability to master the learning environment), and relationship (feeling connected with peers, teacher, and administrators). If these conditions are not met, the student usually experiences academic stress or thinks about dropping out (Yu and Levesquel-Bristol, 2020).

To understand the predictor variables of study satisfaction in the context of the COVID-19 pandemic, the literature highlights the importance of psychological distress, anxiety, and academic self-efficacy because of their impact or the role they play.

Herrera and Rivera (2011) defined psychological distress as a reactive state that involves perceiving discomfort owing to psychological alterations related to perceived stress, depression, anxiety, or demoralization (Liébana-Presa et al., 2014). In this regard, research conducted 10 years ago revealed that a low level of students’ life satisfaction could be predicted as a function of experiencing symptoms related to anxiety, depression, and satisfaction with their department and socioeconomic level (Bulut Serin et al., 2010). Such facts were corroborated during the COVID-19 pandemic by Babar et al. (2021), who found that 41% of students face severe psychological distress and 65% face dissatisfaction with online classes. Under a linear regression model, these data show that psychological distress is a predictor of satisfaction with virtual education.

Flores et al. (2016) were the first to define anxiety as an adaptive response to a stressor. In this regard, studies such as that carried out by Arjanggi and Shanti (2016) concluded that social anxiety generates a negative effect on academic adjustments within the context of the educational experience in first-year university students. Likewise, Abdous (2019) reports that gender, prior online experience, and the feeling of readiness are variables related to feelings of anxiety. Regarding academic self-efficacy, it is understood as the belief the students have in their own capacity and efficiency to carry out tasks in the academic setting (Hechenleitner-Carvallo et al., 2019). In this regard, research such as that conducted by Shen et al. (2013) found that the self-efficacy of online learning accounts for learning satisfaction, a result that is gaining momentum with the finding of Kostagiolas et al. (2019), who conclude that, in effect, there are functional relations between study satisfaction, self-efficacy, and academic performance among undergraduate students.

With that in mind, throughout the years, there has always been an interest in understanding the cognitive and behavioral factors that favor or limit students’ performance in terms of academic requirements in the university context (Contreras et al., 2005; Cabanach et al., 2012; Gutiérrez-García and Landeros-Velázquez, 2018). Therefore, considering the uncovered evidence and the literature gap regarding the study of factors that has predictive power on satisfaction with studies in times of health crisis, determining which variables play an important role in the enjoyment of higher education experiences in the context of the COVID-19 pandemic is required.

In view of the foregoing, this research aims to determine if psychological distress, anxiety, and academic self-efficacy predict study satisfaction among Peruvian university students during the COVID-19 pandemic.

## Materials and Methods

### Study Design

This is a cross-sectional predictive study (Ato et al., 2013) which uses psychological distress, anxiety, and academic self-efficacy as predictor variables, while the criterion variable is study satisfaction of Peruvian university students.

### Study Participants

Non-probability sampling was used. The prior power analysis performed in the G*Power program (Faul et al., 2009), with a small effect size (f2 = 0.15), α = 0.05, and power = 0.95 and with three predictors, indicated that 74 participants were enough to identify the effects. However, the actual sample exceeded that number as 582 university students (58.2% female) participated. Participants attended different Peruvian universities, and their age ranged from 16 to 41 years (M = 21.79; SD = 5.05). From all, 90% were studying at a private university, 39.2% attended the School of Engineering and Architecture, and 36.4% were students from the School of Health Sciences.

The university students evaluated carried out their studies during the COVID-19 pandemic. Therefore, the transition from face-to-face to virtual education led to changes and challenges (Vilela et al., 2021). This led to a lot of worry and pressure because the students had to use a methodology that they were not trained to work with, and, for which, they were unprepared (Suárez et al., 2021). However, since the majority of the cohort studied in private universities, they had more opportunities to access virtual education. Nevertheless, while before the pandemic, the school dropout rate ranged from 15.8 to 17.6%, this rate increased to 18.1–42.6% during the pandemic (Benites, 2021).

### Measures

*Brief Scale of Study Satisfaction (EBSE; Merino-Soto et al., 2017).* This is a brief measurement consisting of three items. It assesses the students’ satisfaction with their way of studying, their academic performance satisfaction, and their global study satisfaction. The items are in Likert-type format, with five response options stating agreement or disagreement with the statements, from “In strong disagreement” to “In strong agreement.” In this study, the EBSE showed good internal consistency (α = 0.87 [CI 95%:0.84–0.88]).

*Generalized Anxiety Disorder Scale-2 (GAD-2; Kroenke et al., 2007).* This is a brief measurement consisting of two items that assess the frequency of occurrence of behaviors linked to the generalized emotional and cognitive expression of anxiety in the last 2 weeks. The items are scaled in Likert-type format, with four response options, from 0 (none) to 3 (almost every day). The version used was adapted to Peruvian Spanish.^1^ For the study, the GAD-2 reported adequate reliability (α = 0.84 [CI 95%:0.81–0.86]).

*Academic Self-Efficacy Scale [EAPESA (for its Spanish acronym); Palenzuela, 1983].* This scale assesses self-efficacy specifically perceived in academic situations. The version used was adapted for university students (Dominguez et al., 2012). It consists of nine items, with four response options, from “Never” to “Always.” The reliability of the EAPESA in this study was α = 0.93 (CI 95%:0.91–0.94).

*Kessler Psychological Distress Scale (K6; Kessler et al., 2003).* This scale consists of six items that assess psychological distress based on two factors, anxiety and depression, through the frequency with which the students experienced non-specific symptoms during the last 30 days. The items are based on the criteria diagnosed for major depression and generalized anxiety disorder from the fourth edition of the Diagnostic and Statistical Manual of Mental Disorders (DSM-IV; APA, 2004). The items are scaled in Likert-type format, with five response options, the values of which go from 0 (Never) to 4 (All the time). The version applied was that used in Peru by Dominguez-Lara and Alarcón-Parco (2020). In this study, the Psychological Distress Scale K6 showed adequate internal consistency (α = 0.84 [CI 95%:0.81–0.86]).

### Study Procedure

The study was approved by the ethics committee of the Universidad Peruana Unión (2020-CEUPeU-00023). Participants were contacted via social media (Facebook and WhatsApp), and the instruments were answered through Google forms. Before completing the instruments, they were required to sign an informed consent that communicated the purpose of the study and the anonymous and voluntary nature of their participation.

### Statistical Analysis

The analysis of data started with a descriptive analysis of the variables: study satisfaction, psychological distress, anxiety, and academic self-efficacy. The students’ *t*-test was used to assess the differences among the variables by gender. Additionally, Cohen’s *d* was used as an effect size (ES) measure to compare two independent groups (Caycho-Rodríguez, 2017a), where values of 0.20, 0.50, and 0.80 express a small, medium, and large ES, respectively (Cohen, 1998; Ferguson, 2009). The analysis of the relation between the study variables was carried out with Pearson’s correlation coefficient, calculating the correlations’ effect size based on the correlation coefficient value (≥0.20: minimum recommended; ≥0.50: moderate; ≥0.80: strong) and their respective confidence intervals (Caycho-Rodríguez, 2017b). Lastly, a regression model was estimated by calculating the ES based on the determination coefficient (*R*^2^) and its confidence intervals, where the values ≥ 0.02, ≥ 0.13, and ≥ 0.26 indicate a small, average, and large ME, respectively (Caycho-Rodríguez, 2017c,d). The statistical software SPSS, version 24.0, was used to conduct the statistical studies.

## Results

### Descriptive Analyses

Table 1 shows that the skewness and kurtosis coefficients are below 1.5, this being an adequate range (Pérez and Medrano, 2010).

**TABLE 1:** Descriptive analyses of study satisfaction, psychological distress, anxiety, and academic self-efficacy.

Variables	M	SD	Sc	K
Study satisfaction	9.35	2.877	–0.179	–0.471
Psychological distress	13.32	4.688	0.730	0.494
Anxiety	3.84	1.711	0.772	–0.122
Academic self-efficacy	25.64	5.903	–0.059	–0.488

*M, Mean; SD,Standard Deviation; Sc,Skewness Coefficient; K,Kurtosis Coefficient.*

### Differences Between Study Satisfaction, Psychological Distress, Anxiety, and Academic Self-Efficacy by Gender

As can be observed in Table 2, the students’ *t*-test for independent samples indicates that there are no significant differences in study satisfaction between men and women. On the contrary, significant differences were found with respect to psychological distress (*t* = 1,110, *p* = 0.267), anxiety (*t* = −3,097, *p* = 0.002), and academic self-efficacy (*t* = −3,865, *p* = 0.000) between men and women. The analysis of mean values shows that women present higher levels of psychological distress and anxiety, whereas men show greater scores in academic self-efficacy. With respect to the effect size calculated by means of Cohen’s *d*, it is observed that the ES for the psychological distress variable is within the limit (*d* = 0.20), by no means negligible for the anxiety variable (*d* = 0.32), and good for academic self-efficacy (*d* = 0.57).

**TABLE 2:** Study satisfaction, psychological distress, anxiety, and academic self-efficacy between men and women.

	Men		Women		*t*	*p*	*d*
	M	SD	M	SD			
Study satisfaction	9.51	3.003	9.24	2.782	1.11	0.267	–0.09
Psychological distress	12.62	4.633	13.83	4.669	–3.097	0.002	0.26
Anxiety	3.52	1.672	4.07	1.704	–3.865	0.000	0.32
Academic self-efficacy	26.59	6.055	24.97	5.704	3.295	0.001	0.57

### Correlation Between Study Satisfaction, Psychological Distress, Anxiety, and Academic Self-Efficacy

Table 3 shows Pearson’s correlations between study satisfaction, psychological distress, anxiety, and academic self-efficacy. A statistically significant correlation between the study variables (*p* < 0.01) can be observed.

**TABLE 3:** Correlation between study satisfaction, psychological distress, anxiety, and academic self-efficacy.

	Study satisfaction	Psychological distress	Anxiety	Academic self-efficacy
Study satisfaction	1			
Psychological distress	−0.178**	1		
Anxiety	−0.122**	0.580**	1	
Academic self-efficacy	0.429**	−0.348**	−0.192**	1

**Significant at the 0.01 (bilateral) level.

### Study Satisfaction Prediction

To determine the variables that better predict study satisfaction, a multiple regression study was carried out. The psychological distress, anxiety, and academic self-efficacy variables were introduced into this study. From these, academic self-efficacy turned out to be the predictor variable. Table 4 shows the adjusted *R*, *R*^2^, *R*^2^ multiple correlation coefficients, the standard error of estimate (EE), and the ANOVA *F*-value.

**TABLE 4:** Linear correlation coefficients *R*, *R*^2^, adjusted *R*^2^, EE, and *F*.

Model	*R*	*R* ^2^	Adjusted *R*^2^	EE	*F*	Foll.
1	0.429^a^	0.184	0.183	2.601	131.005	0.000^b^

*^a^Predictor variables: (Constant), Academic self-efficacy.*

*^b^Dependent variable: Study satisfaction.*

As shown in Table 4, the coefficient of determination *R*^2^ = 0.184 indicates that the academic self-efficacy variable accounts for 18.4% of the total criterion variable, study satisfaction. A greater value of the multiple determination coefficient indicates a greater explanatory power of the regression equation and, therefore, greater power of prediction of the dependent variable. The adjusted *R*^2^ accounts for the percentage of 18.3%. The ANOVA *F*-value (*F* = 131.005, *p* = 0.000) indicates that there is a significant linear relationship between the academic self-efficacy variable (predictor) and the study satisfaction variable (criterion).

Table 5 shows the unstandardized regression coefficients (B), the standardized regression coefficients (β), and the statistical coefficients related to the predictor variable. Coefficient β indicates that academic self-efficacy (predictor variable) significantly predicts study satisfaction (criterion variable). The T-value of beta regression coefficients of the predictor variable has been found to be highly significant (*p* < 0.01).

**TABLE 5:** Multiple regression coefficients B (unstandardized), β (standardized), and *t*-test.

Model	B	EE	β	*t*	Foll.
1(Constant)	3.989	0.481		8.293	0.000
Academic self-efficacy^a^	0.209	0.018	0.429	11.446	0.000

*^a^Dependent variable: Study satisfaction.*

## Discussion

The COVID-19 outbreak has become a global problem with its impact exceeding the organic clinical manifestations related to this disease, with consequences in the mental health of the population being one of the emerging concerns requiring prompt and effective response from the field of psychology. In this situation, this article aimed at assessing the predictive role of psychological distress, anxiety, and academic self-efficacy with regard to study satisfaction among Peruvian university students during the COVID-19 pandemic. To do so, linear regression models were tested, and different comparative and correlational analyses were conducted.

As for the gender-based comparative analyses, the results of the study have shown that women presented higher scores for psychological distress than men, with said differences being significant. Similarly, other studies found that psychological distress is greater for women, as they have reported recent adverse events and difficulties in adapting to the academic environment more often than men (Verger et al., 2009). In other words, even with evidence of women surpassing men in some spheres, they are more prone to suffering from psychological distress (Adlaf et al., 2001; Pomerantz et al., 2002; Eisenbeck et al., 2019).

Regarding anxiety, significant differences were found in terms of gender, as women showed signs of anxiety more frequently than men. Other studies have reported similar results in different countries, both in the pre-pandemic context (Rahafar et al., 2016; Tran et al., 2018) and in the pandemic scenario (Bigalke et al., 2020; Debowska et al., 2020; Wang and Zhao, 2020; Burkova et al., 2021; Rodriguez-Besteiro et al., 2021). Additionally, there is evidence that the influence and intensity of academic anxiety are higher in women compared to men (Bhansali and Trivedi, 2008). Consequently, the fact that women present more indicators of psychological distress and anxiety than men possibly precedes the pandemic, but the pandemic context may increase these divergences (Debowska et al., 2020).

As for academic self-efficacy, this study shows significant gender-related differences. Women have lower academic self-efficacy levels than men, and these results are in line with those reported in other studies (Bondy et al., 2017; Yilmaz, 2017; Ryan and Poole, 2019). In other research reported that even when said differences are significant, the effect size is quite small (Huang, 2013). However, in this study, the effect size was large. Mohammadyari (2012) states that these differences may be the result of men’s ability and the confidence they have on their ability in contrast with women. Another explanation is that the women’s self-efficacy decreases as they progress through middle and secondary school (Assouline et al., 2020), which makes them change their focus toward non-academic objectives (Brown et al., 2019). Also, receiving feedback is considered to increase self-efficacy levels among women, which is in line with the idea that said differences are the result of men feeling more confident about their own skills than women (Bong, 1999). Conversely, given the evidence that anxiety is negatively correlated with self-efficacy (Chan, 2002), the high scores on the anxiety scale found in the female sample in the pandemic context may have had a negative impact on their beliefs about their abilities and academic performance; that is, it may have affected their self-efficacy.

With regard to study satisfaction, results show that the differences found between the answers provided by men and women are not significant. With that in mind, inconsistent results were found in the literature. This way, on the one hand, research indicates that the understanding of the variables associated with academic satisfaction may vary based on gender (Parahoo et al., 2013), while, on the other hand, studies found no evidence to corroborate the assumption that the women’s study satisfaction is different from their classmates (Thege, 2014). In any case, it is therefore necessary to address this subject from the perspective of other methodological aspects.

At the correlational level, this study also shows a significant correlation between academic self-efficacy and study satisfaction. In this sense, other research has demonstrated that both the variables are significantly related. Sivandani et al. (2013) even assert the existence of a positive influence of study satisfaction on self-efficacy (Zhen et al., 2017). However, it has also been reported that said variables do not relate to each other in the context of an environment open to remote learning (Coetzee and Oosthuizen, 2012). Conversely, it could be observed that the highest anxiety levels are significantly related to low levels of study satisfaction. Although said links are weak, evidence has shown that self-efficacy is an important predictor of the main effects of anxiety issues (Chan, 2002).

The results also show that psychological distress and anxiety are negatively related to study satisfaction. Despite their weakness, these have been considered significant at a statistical level. Although some studies show that students with lower anxiety levels are more satisfied than those showing higher levels of anxiety (Bolliger and Halupa, 2012), this is not the case of the relationship between psychological distress and study satisfaction. Thus far, we know that students’ psychological distress may influence other variables such as professional development, and it seems to negatively affect academic performance and contribute to academic dishonesty and substance abuse (Lepp et al., 2014). Several studies show that psychological distress is significantly associated with an increased risk of developing anxiety (Verger et al., 2009), and, in turn, anxiety, in the context of exams, is a significant predictor of psychological distress (Rajiah et al., 2014). Further, conversely, there is evidence of the fact that anxiety contributes to low well-being and academic performance levels (Leung et al., 2000; Antaramian, 2017; McIntyre et al., 2018).

In response to the main objective of the study, after including all the variables within a predictive model of study satisfaction, it could be observed that academic self-efficacy has a higher predictive value than psychological distress and anxiety. Thus, some studies highlight the predictive role of self-efficacy in study satisfaction and, in turn, indicate that the combination of self-efficacy and study satisfaction may be an essential mechanism to improve the academic performance of students (Chemers et al., 2001; Kostagiolas et al., 2019). These results are consistent with those of studies that point to self-efficacy as a predictor variable of academic performance in university students in the context of the pandemic (Talsma et al., 2021). A potential interpretation is that self-efficacy plays a relevant role in terms of satisfaction with studies and other variables related to academic performance of university students in a context characterized by uncertainty; therefore, it constitutes a protective variable against the risks of academic maladaptation caused by the COVID-19 pandemic. All this leads to the need to conduct further studies that provide further evidence on how said variables predict study satisfaction, as well as consider other variables, such as social support and academic performance, so they may help understand not only the predictable role but also the predictive function of study satisfaction.

The limitations of this research include, on the one hand, the exclusive use of self-reporting for the assessment of the study variables that may lead to biases related to the perception of the behavior itself. Additionally, the cross-sectional design limits the evaluation of causality between the predictor variables and the dependent variable. Future studies should address this problem using other causal or longitudinal designs (Ato et al., 2013). However, the absence of differentiated samples based on the level of responses of the educational institutions in implementing virtual teaching scenarios and the use of non-probabilistic sampling procedures limit the possibility of generalizing the results to the population. Despite the difficulties noted, the findings presented here are relevant as an approach to the study phenomenon in the broader context of higher education and seek to stimulate the development of research that delves into the problems arising from the new educational context, the impact of self-efficacy in other spheres of academic life, and the mental health of university students. Another limitation may be the presence of common method variance bias because both the predictor and outcome variables were self-reported in a cross-sectional survey. However, it is important to mention that recent studies based on statistical simulations indicated that the phenomenon must be very high (approximately more than 70%) to unduly inflate the correlations (Fuller et al., 2016). Similarly, other authors have examined the presence of common method variance in their data and found only low levels of variance bias (Schaller et al., 2015).

Thus, the significance of this research is that it highlights the importance of understanding the mechanisms that predict study satisfaction among university students to enable the implementation of effective and timely mental health policies and interventions aimed at improving academic self-efficacy and optimizing the learning experience of university students. Additionally, based on the results of this study, an increase in academic self-efficacy would translate into a decrease in psychological distress and anxiety. This, and the adoption of a comprehensive approach to mental healthcare and the welfare of the university community in a context that is particularly challenging, is essential to address the adverse effects of the pandemic.

## Data Availability Statement

The raw data supporting the conclusions of this article will be made available by the authors, without undue reservation.

## Ethics Statement

The studies involving human participants were reviewed and approved by Universidad Peruana Unión. Written informed consent to participate in this study was provided by the participants’ legal guardian/next of kin.

## Author Contributions

RCE, OM-B, and PRM conceived and designed the experiments, performed the experiments, analyzed and interpreted the data, and wrote the manuscript. TC-R and SL-H contributed reagents, materials, analysis tools, or data and wrote the manuscript. All authors contributed to the article and approved the submitted version.

## Conflict of Interest

The authors declare that the research was conducted in the absence of any commercial or financial relationships that could be construed as a potential conflict of interest.

## Publisher’s Note

All claims expressed in this article are solely those of the authors and do not necessarily represent those of their affiliated organizations, or those of the publisher, the editors and the reviewers. Any product that may be evaluated in this article, or claim that may be made by its manufacturer, is not guaranteed or endorsed by the publisher.

## Footnotes

1
https://www.phqscreeners.com
